# Digest before Ingest: Early Recruitment of Membrane-bound DNaseX to Phagocytic Cups in Macrophages

**DOI:** 10.64898/2026.02.23.707500

**Published:** 2026-02-24

**Authors:** Arghajit Pyne, Vivek Pandey, Subhankar Kundu, Sachie Ikegami, Xuefeng Wang

## Abstract

Macrophages engulf and degrade pathogens and cellular debris through phagocytosis. The degradation process was generally believed to occur only after phagosome internalization and maturation. Here, we report an early DNase activity at the nascent phagocytic cup (PC) prior to its closure. Using a fluorescent DNase sensor, we revealed rapid and ubiquitous DNase activity upon PC formation across various macrophage types. We further identified the responsible enzyme as the membrane-bound DNaseX, which is constitutively recruited to the PC during PC formation. Although F-actin polymerization is dispensable for DNaseX recruitment, it is essential for its enzymatic activity, likely by promoting physical engagement of DNaseX with solid DNA materials. Functionally, we show that macrophages degrade extracellular DNA (eDNA) within bacterial biofilms through direct physical contact, clearing the eDNA structures without internalization. These findings reveal a previously unrecognized DNA degradation mechanism operating at the macrophage membrane, suitable for degrading bulky eDNA materials which cannot be directly internalized by macrophages.

## Full Text Availability

The license terms selected by the author(s) for this preprint version do not permit archiving in PMC. The full text is available from the preprint server.

